# Percutaneous Closure of Patent Foramen Ovale and Atrial Septal Defect in Athletes: A Study With a Long‐Term Follow‐Up

**DOI:** 10.1111/sms.70116

**Published:** 2025-08-08

**Authors:** Flavio D'Ascenzi, Vincenzo Minasi, Guglielmo Leonardo Manfredi, Gian Luca Ragazzoni, Luna Cavigli, Giulia Elena Mandoli, Maria Concetta Pastore, Marta Focardi, Matteo Cameli, Serafina Valente, Massimo Fineschi

**Affiliations:** ^1^ Department of Medical Biotechnologies, Sports Cardiology and Rehab Unit University of Siena Siena Italy; ^2^ Division of Cardiology, Policlinico Tor Vergata University of Rome Tor Vergata Rome Italy; ^3^ Division of Cardiology, Department of Medical Biotechnologies University of Siena Siena Italy; ^4^ Division of Interventional Cardiology University Hospital Santa Maria Alle Scotte Siena Italy

**Keywords:** athletes, bleeding, dislocation, patent foramen ovale, percutaneous closure, traumatic sports

## Abstract

Patent foramen ovale (PFO) and atrial septal defect (ASD) percutaneous closure is routinely performed. However, data on long‐term safety and efficacy in athletes are lacking. This study with a long‐term follow‐up investigated the safety and efficacy of percutaneous PFO/ASD closure in athletes compared to non‐athletes, focusing on the impact of sports participation on complications. The study included individuals < 50 years old undergoing percutaneous PFO/ASD closure. Athletes practiced sports for ≥ 4 h/week for ≥ 2 consecutive years before and after the procedure. Periprocedural complications, device dislocation, and bleeding events were assessed during a long‐term follow‐up (mean 10.6 ± 5.1 years). Echocardiography, including transcranial Doppler bubble test, was performed before discharge, at 1 and 3 months, and yearly thereafter. Seventy‐six patients (36 athletes and 40 non‐athletes) were included. Fourteen patients practiced sports at risk of trauma, and no differences in periprocedural complications were observed between the two groups. During the follow‐up, no device dislocation was observed in athletes. A single major bleeding occurred in a non‐athlete during dual antiplatelet therapy (DAPT). Eight minor bleedings occurred (4 during DAPT, 4 during single antiplatelet therapy—SAPT), with no significant difference between athletes and non‐athletes (*p* = 0.23 and *p* = 0.79, respectively). One minor sports‐related bleeding (muscular hematoma) occurred during SAPT in an athlete practicing kickboxing. Percutaneous PFO/ASD closure is safe in athletes, with no differences in complication rates between athletes and non‐athletes observed during long‐term follow‐up (> 10 years). Sports participation did not significantly increase the bleeding risk, even in athletes practicing sports at risk of trauma.

## 1 Introduction

Congenital interatrial septal defects are one of the most frequent cardiac anomalies. Among these, patent foramen ovale (PFO) and atrial septal defects (ASDs) are the most common. ASD has a low prevalence, occurring in approximately 1:1500 live births and is rarely asymptomatic; it may require percutaneous or surgical closure at a young age [1, 2], especially when of considerable size, due to right ventricular volume overload and, eventually, pulmonary hypertension [3]. In addition, it may potentially lead to ischemic strokes. Notably, a rapid reverse remodeling of the right ventricle has been described in competitive athletes after ASD closure [4]. Conversely, PFO is a frequent condition caused by a fusion failure between the septum primum and septum secundum at the anterosuperior border of the fossa ovalis after birth. It is estimated that about 15%–35% of healthy adults have a PFO [5], being asymptomatic in most cases. Nonetheless, it may play a pathogenic role in various clinical syndromes, particularly in paradoxical embolism, whose most significant consequence is cryptogenic stroke [6, 7]. Relevant studies on PFO were published in 2013 [8, 9, 10] and subsequent research activity produced substantial findings [11], culminating in 2019 with the release of the European position paper [12] aimed at standardizing the management of patients with left‐sided thromboembolism associated with PFO. Given the absence of clear guidance regarding PFO management in the general population, it is not surprising that the body of evidence regarding specific subpopulations such as athletes, remains limited. However, these patients deserve more in‐depth consideration of the clinical indications and safety for closure, with the need for therapeutic management tailored to their unique circumstances. This study aimed to assess the safety and efficacy of percutaneous closure of PFOs and ASDs in competitive athletes compared to sedentary individuals and to evaluate the impact of sports practice on the rate of potential complications during long‐term follow‐up.

## 2 Methods

### 2.1 Study Design

In this retrospective study, we consecutively enrolled patients with PFO or ASD who underwent percutaneous closure in the Division of Interventional Cardiology at the University Hospital in Siena. Patients aged over 50 years at the time of closure were excluded from the study. After the closure, the echocardiographic evaluation was performed before discharge, at 1‐ and 3‐month follow‐up including a transcranial Doppler bubble test [13] to identify residual shunts. A yearly echocardiographic evaluation and a telephone interview were then conducted after the first year to assess the patients' well‐being, the occurrence of any complications, their current therapy, and whether they were participating in competitive or non‐competitive sports.

### 2.2 Study Group

After the enrollment, according to the clinical data available and collected as per protocol in our department, the study population was divided into two groups: competitive athletes and sedentary individuals. Patients who practiced sports before the procedure and resumed participation as soon as possible, practicing at least 4 h per week for a minimum of 2 consecutive years, irrespective of their involvement in competitions, were included in the athletes' group. This study cohort did not include professional athletes. In competitive athletes, sports disciplines were classified according to the ESC Sports Cardiology Guidelines (14) into four types: (1) skill, that is, golf, table tennis…; (2) power, that is, wrestling, boxing…; (3) mixed, that is, soccer, tennis, volleyball…; and (4) endurance, that is, cycling, triathlon… Sports disciplines were also divided into sports at risk of trauma and non‐traumatic sports. It should be noted that there is no single formalized classification focused explicitly on trauma risk from collisions in all types of sports activities globally recognized across all disciplines or governing bodies in the field of sports medicine or safety regulations at the present time; researchers use various frameworks to assess injury risks associated with different categories of physical activities like collision/contact versus non‐contact ones based upon empirical evidence available up until now. Therefore, we behaved accordingly based on the studies in the scientific literature [14, 15, 16, 17]. Sedentary patients who were not engaged in any exercise activities were defined as non‐athletes. The demographic characteristics of the study population, cardiovascular risk factors, characteristics of the implanted devices, periprocedural and follow‐up complications, the duration of dual and single antiplatelet therapy during the follow‐up, the type and volume of sports practiced, and complications during sports related to the presence of the device or therapy were collected.

The study was drafted and approved by the local ethical committee of the University Hospital in Siena (prot. Vers. 2.0/2020).

### 2.3 Periprocedural Complications and Follow‐Up

Periprocedural and post‐procedural complications were defined according to their occurrence before and after 24 h, respectively. The complications were categorized as major bleeding, minor bleeding, and device dislocation. Major bleedings were defined as bleedings severe enough to cause hemodynamic shock, requiring hospitalization in a critical anatomical site (e.g., the brain) or requiring two or more blood transfusions; minor bleedings were all the others [18, 19]. Device dislocation was defined as a displacement of the device from its original site and potential migration [20]. Complications were defined according to their definition in the scientific literature [21, 22].

## 3 Results

### 3.1 Demographic Characteristics and Periprocedural Data

The study population consisted of 76 patients: 36 athletes (59% male and 41% female, with a mean age at defect closure of 33 ± 8 years) and 40 non‐athletes (35% male and 65% female, with a mean age at defect closure of 37 ± 7 years). The demographic characteristics are reported in Table 1. Power sports (47%) were the most practised among athletes, followed by mixed sports (36%). Thirty‐nine per cent of athletes practised contact sports (31%); see Table S1. Most participants underwent PFO closure (87%), with no significant differences between the groups (83% in athletes vs. 90% in non‐athletes). At the preprocedural transesophageal echocardiogram, we found 7 tunneled PFOs (4 sedentary individuals and 3 athletes), 1 case of an ASD associated with PFO in a sedentary individual, and 1 filamentous Eustachian valve in a sedentary subject. Procedural characteristics, medical therapy, and periprocedural complications related to defect closure are shown in Tables 2 and 3. No differences were observed between athletes and non‐athletes in terms of periprocedural complications. The duration of dual antiplatelet therapy (DAPT) was similar in both groups, whereas the duration of SAPT was longer in non‐athletes than in athletes (52.6 ± 70.9 vs. 32.4 ± 49.1 months, respectively), although there was no significant difference between the two groups.

**TABLE 1:** Demographic characteristics of the study population.

	All	Athletes	Non‐athletes	*p*
Age, years	35 ± 8	33 ± 8	36 ± 7	< 0.05
Male sex, no. (%)	35 (46)	21 (60)	14 (40)	0.09
Female sex, no. (%)	41 (54)	15 (37)	26 (64)	0.09
Weight, kg	69.3 ± 14.6	70.3 ± 12.7	68.45 ± 16.3	0.65
Height, cm	170.3 ± 9.0	172.8 ± 7.8	168.9 ± 9.9	0.24
BSA	1.8 ± 0.2	1.8 ± 0.3	1.8 ± 0.2	0.47
BMI	23.9 ± 4.3	23.7 ± 3.4	24.0 ± 5.1	0.81
CV risk factors				
Hypertension, no. (%)	8 (10)	2 (5)	6 (15)	0.18
Dyslipidemia, no. (%)	6 (8)	2 (5)	4 (10)	0.48
Diabetes mellitus, no. (%)	0 (0)	0 (0)	0 (0)	NA
Current smokers, no. (%)	16 (21)	5 (14)	11 (27)	0.83
Type of sport				
Skill, no. (%)	NA	2 (6)	NA	
Power, no. (%)	NA	17 (47)	NA	
Mixed, no. (%)	NA	13 (36)	NA	
Endurance, no. (%)	NA	4 (11)	NA	
Echocardiographic features				
Ejection fraction	61.2 ± 3.2	60.9 ± 3.5	61.4 ± 2.9	0.81
LV end diastolic diameter	46.5 ± 4.9	46.5 ± 6.0	46.4 ± 3.7	0.23
TAPSE	23.1 ± 3.2	24.2 ± 3.1	22.1 ± 3.1	0.88
PAPs	24.0 ± 4.1	23.3 ± 4.2	24.5 ± 4.1	0.4
Risk scores				
RoPE score	7.3 ± 1.1	7.5 ± 1.1	7.2 ± 1.1	0.85
HAS‐BLED	0.95 ± 1.1	0.9 ± 1.0	1.0 ± 1.1	0.21

*Note:* *p* value: comparison between athletes and non‐athletes.

Abbreviations: BMI, body mass index; BSA, body surface area; CV, cardiovascular risk; NA, not applicable.

**TABLE 2:** Device characteristics and medical therapy.

	All	Athletes	Non‐athletes	*p*
Type of congenital heart disease				
PFO, no. (%)	66 (87)	30 (83)	36 (90)	0.39
ASD, no. (%)	10 (13)	6 (17)	4 (10)	
Type of device				
Amplatzer, no. (%)	61 (80)	29 (80)	32 (80)	0.65
Figulla flex, no. (%)	5 (7)	2 (6)	3 (8)	
Occlutech ASD occluder, no. (%)	10 (13)	5 (14)	5 (12)	
Post‐procedural antiplatelet therapy				
Only SAPT, no. (%)	7 (9)	4 (11)	3 (8)	0.62
DAPT, no. (%)	69 (91)	32 (89)	37 (92)	0.59
DAPT average duration, months	1.62 ± 1.5	1.6 ± 1.5	1.7 ± 1.5	0.88
SAPT average duration, months	43.0 ± 61.9	32.4 ± 49.1	52.6 ± 70.9	0.16

*Note:* *p* value for comparison between athletes and non‐athletes.

Abbreviations: ASD, atrial septal defects; DAPT, dual antiplatelet therapy; PFO, patent foramen ovale; SAPT, single antiplatelet therapy.

**TABLE 3:** Peri‐procedural complications.

	All	Athletes	Non‐athletes	*p*
Intra‐operative or early complication (< 24 h), no. (%)	7 (9)	3 (8)	4 (10)	0.18
Pericardial effusion (non‐tamponade), no. (%)	3 (4)	1 (3)	2 (5)	
Laryngospasm, no. (%)	1 (1)	1 (3)	0 (0)	
Blurred vision, no. (%)	1 (1)	0 (0)	1 (2)	
Supraventricular tachyarrhythmia, no. (%)	1 (1)	1 (3)	0 (0)	
Atrioventricular block (II grade), no. (%)	1 (1)	0 (0)	1 (2)	

*Note:* *p* value for comparison between athletes and non‐athletes.

### 3.2 Clinical Long‐Term Follow‐Up

**FIGURE 1:**
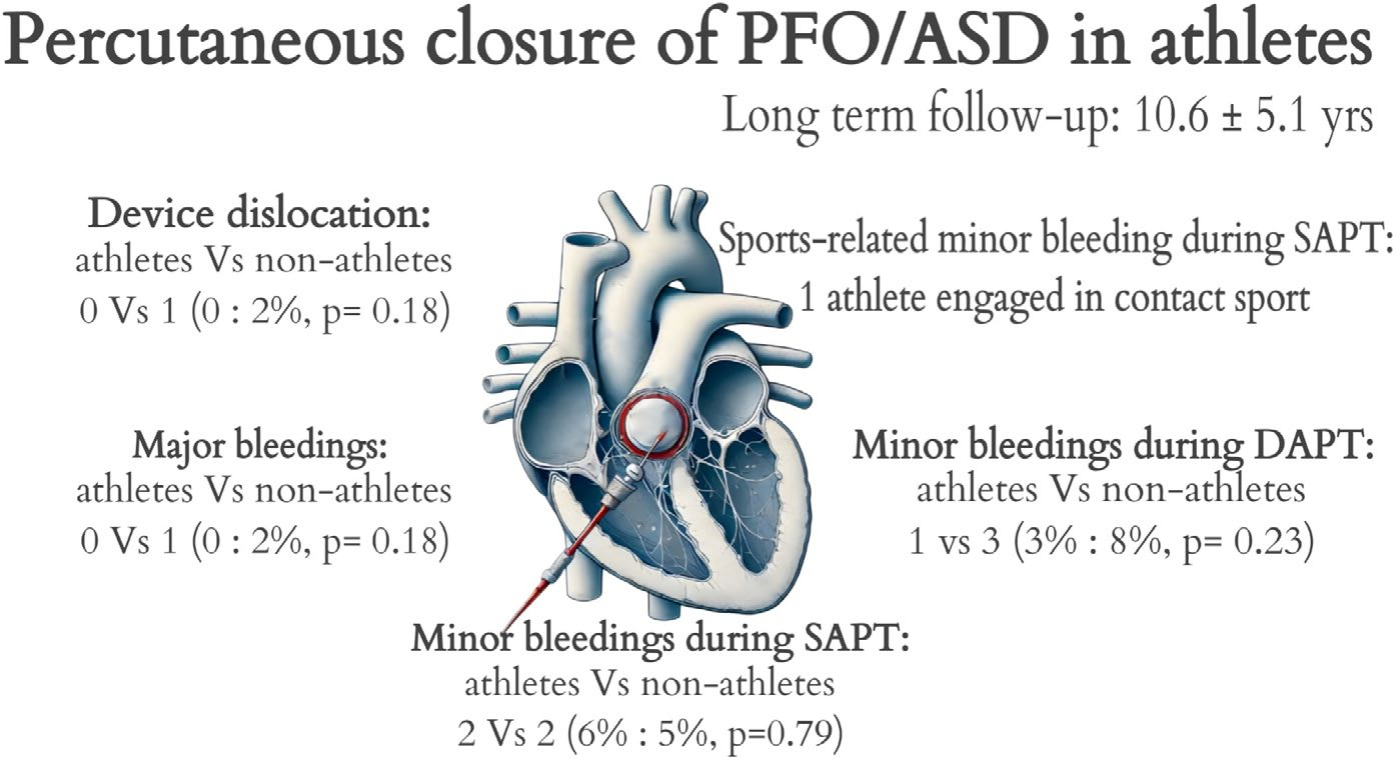
Central illustration summarizing the main findings of the study.

The duration of clinical follow‐up was 10.6 ± 5.1 years. Adverse events that occurred during the follow‐up are reported in Table 4. During the follow‐up, one device dislocation was observed in the entire study population, occurring in a sedentary patient, probably due to the device size or the unfavorable anatomy of the PFO, and it was resolved by removing the device from the right femoral artery through a vascular surgery procedure. No cases of device thrombosis were observed during the follow‐up. In terms of bleeding, one major event occurred in the non‐athletic group while the patient was on DAPT; intracranial bleeding was identified with brain magnetic resonance imaging and resolved spontaneously with the suspension of antiplatelet therapy. Eight minor bleeding events occurred during the follow‐up, four during DAPT and four during SAPT, with no difference between athletes and non‐athletes. In the athletic group, two minor bleedings were reported, and only one was related to sports practice and occurred in a patient practicing a sport at risk of trauma (kickboxing) while on SAPT with acetylsalicylic acid: a muscular hematoma spontaneously reabsorbed in a few days. Five minor events occurred in the non‐athletic group. A central illustration summarizing the main findings of this study is reported in Figure 1.

**TABLE 4:** Adverse events during a long‐term follow‐up.

	All	Athletes	Non‐athletes	*p*
Duration of clinical follow‐up, years	10.6 ± 5.1	11.1 ± 5.0	10.2 ± 5.3	0.50
Echocardiographic follow‐up, years	8.0 ± 5.1	8.4 ± 5.3	7.7 ± 5.0	0.24
Years of sports practise after the intervention	NA	9.1 ± 5.1	NA	NA
Device dislocation during follow‐up, no. (%)	1 (1)	0 (0)	1 (2)	0.18
Major bleeding during DAPT, no. (%)	1 (1)	0 (0)	1 (2)	0.18
Major bleeding during SAPT, no. (%)	0 (0)	0 (0)	0 (0)	NA
Minor bleeding during DAPT, no. (%)	4 (5)	1 (3)	3 (8)	0.23
Hematoma, no. (%)	2 (3)	1 (3)	1 (2)	
Gingival bleeding, no. (%)	2 (3)	0 (0)	2 (5)	
Minor bleeding during SAPT, no. (%)	4 (5)	2 (6)	2 (5)	0.79
Hematoma, no. (%)	3 (4)	1 (3)	2 (5)	
Gingival bleeding, no. (%)	1 (1)	1 (3)	0 (0)	
Sports‐related major bleeding	NA	0 (0)	NA	NA
Sports‐related minor bleeding	NA	1 (3)	NA	NA
Residual shunt, no. (%)	20 (26)	10 (28)	10 (25)	0.79
Minimal, no. (%)	5 (7)	5 (14)	0 (0)	
Mild, no. (%)	12 (16)	2 (6)	10 (25)	
Moderate, no. (%)	3 (4)	3 (8)	0 (0)	
Severe, no. (%)	0 (0)	0 (0)	0 (0)	
TIA or stroke, no. (%)	0 (0)	0 (0)	0 (0)	NA
Persistence of neurological symptoms, no. (%)	4 (5)	1 (3)	3 (8)	0.23
Atrial fibrillation or flutter, no. (%)	1 (1)	0 (0)	1 (2)	0.18
Death, no. (%)	0 (0)	0 (0)	0 (0)	NA

*Note:* *p* value for comparison between athletes and non‐athletes.

Abbreviations: DAPT, dual antiplatelet therapy; NA, not applicable; SAPT, single antiplatelet therapy; TIA, transient ischemic attack.

## 4 Discussion

The percutaneous closure of PFO or ASD is a relatively common interventional procedure. Despite the number of procedures performed worldwide, areas of uncertainty exist, particularly in specific populations such as athletes, regarding the management of therapy, device dislocation, and the rate of post‐procedural complications during follow‐up. Our study aimed to assess the safety and efficacy of percutaneous closure of PFOs and ASDs in athletes compared to sedentary individuals, and to evaluate the impact of sports practice on the rate of potential complications during long‐term follow‐up. The main findings are: (i) no differences were demonstrated between athletes and non‐athletes concerning periprocedural and post‐procedural complications, and no device dislocation was observed in athletes during a long‐term follow‐up, despite the practice of contact sports; (ii) major bleedings did not differ between the two groups and no sports‐related major bleeding was observed in athletes returning to play, in a long‐term follow‐up of sports practice; only one minor bleeding was observed in one athlete practicing contact sports, during SAPT, which spontaneously and rapidly resolved.

### 4.1 Periprocedural Complications and Device Dislocation During a Long‐Term Follow‐Up

This study did not identify any difference in periprocedural complications between athletes and non‐athletes. The migration of a PFO closure device is a rare yet significant complication [20]. It is often associated with device‐related issues (e.g., improper sizing or inaccurate deployment) or anatomical factors (e.g., the presence of an atrial septal aneurysm or hypermobility of the septum) [23]. From a therapeutic perspective, a percutaneous endovascular retrieval may be undertaken. When not feasible or effective, surgical intervention may be required [24, 25]. During the follow‐up, we observed one device dislocation (1.3% of the overall population) that occurred in the non‐athletic group, which is in agreement with previous studies reporting a dislocation rate of around 1% [26]. The event occurred within 3 months after the procedure: the patient was admitted to the Emergency Department because of dyspnea; device dislocation was confirmed, and a vascular surgical intervention was needed to successfully remove the device located in the right common femoral artery. On the contrary, in athletes practicing sports, no device dislocations were observed during a long‐term follow‐up, although 14 of them practiced sports at risk of trauma (39% of the athletic population). This is an important finding because, despite rare and only anecdotal reports in the literature, concerns have historically been raised regarding device dislocation in competitive athletes treated with PFO/DIA occluding devices. In the scientific literature, a case report is frequently cited involving a middle‐aged man, who suffered device dislocation while lifting a heavy load during high‐intensity isometric exertion, with subsequent cardiac perforation. This reported case has raised concerns about the safety of physical activity, particularly isometric exercises, in subjects with closure devices [27]. On the contrary, the present findings confirmed that, in a long‐term follow‐up, device complications were not observed in athletes including those practicing sports at risk of bodily collision or isometric activities.

#### 4.1.1 Major and Minor Bleedings and Sports‐Related Events During a Long‐Term Follow‐Up

One of the main objectives of this study was to assess whether athletes undergoing PFO or ASD percutaneous closure faced a higher risk of bleeding during a long‐term follow‐up. Athletes taking antiplatelet therapy are usually considered at higher risk of bleeding, particularly if they are engaged in sports at risk of bodily collision. Unfortunately, few data addressing the bleeding risk in athletes on antiplatelet therapy are available, particularly after PFO or ASD percutaneous closure, and studies with long‐term follow‐up are lacking. In this study, we demonstrated that the bleeding risk is negligible in athletes, particularly when on SAPT, and clinically relevant sports‐related bleedings were not reported after more than 10 years of sports practice, even if some athletes practiced sports at risk of trauma such as football, rugby, and basketball, disciplines with a well‐known risk of bodily collision [28]. Major bleeding occurred in only one non‐athletic patient during the follow‐up, and no substantial differences in minor bleeding were observed between athletes and non‐athletes. Previous studies have demonstrated a rate of major bleeding around 1%–2%, consistent with the findings in our study [22]. These studies enrolled a higher number of patients with an older average age compared to our population, and consequently, a greater prevalence of cardiovascular risk factors. Furthermore, the average follow‐up was significantly shorter compared to the long‐term data collection performed in this study.

In the group of athletes who continue to practice sports during the follow‐up (mean duration = 11.1 ± 5.0 years), only three minor bleeding events were reported in athletes: one during DAPT and three during SAPT. Only one patient (3% of the athletic population) experienced a sport‐related hematoma (practising kickboxing) during SAPT; the muscular hematoma resolved spontaneously after a few days. In all other cases, the bleeding events were unrelated to participation in sports. Notably, sport‐related bleeds have been assessed only during SAPT; indeed, according to national recommendations and in agreement with international guidelines [29, 30], athletes in DAPT were disqualified from competitions for all sports and also from training in contact sports or sports at risk of trauma until the switch to SAPT. Our findings suggest that, although SAPT slightly increases the risk of bleeding, these incidents are unrelated to sports involvement, and athletes can safely participate in sports during SAPT including those at risk of bodily collision. However, particularly in the case of sports at risk of trauma, careful management and sport‐specific considerations are essential to mitigate the heightened bleeding risks, and the duration of SAPT should be shortened when possible. Indeed, in our study, the absolute duration of SAPT was shorter in athletes compared to sedentary individuals. Notably, although reducing DAPT and SAPT duration is essential to ensure a fast and safe return to sports practice, this should be done only after carefully evaluating personal thrombotic and bleeding risks. In this context, it is essential to inform patients of the potential risks associated with a shortened DAPT and the possible drawbacks, allowing for informed shared decision‐making.

#### 4.1.2 Post‐Closure Atrial Fibrillation (AF)

In this study, we found only 1 case (1% of the overall population) of post‐closure atrial fibrillation (AF) in the non‐athletic group, representing 2% of the cases in this group. AF is a common, though often transient, complication after PFO closure (overall ≈approximately 5% in previous studies), with incidence varying based on the monitoring procedure and usually peaking early post‐procedure [31]. Specific devices, male sex, and larger device sizes increase this risk [32]; despite being generally benign, personalized management is key, but more research is needed to unbundle the topic.

According to the literature, there is a paradoxically greater AF incidence in athletes, particularly middle‐aged male endurance athletes, attributed to different acute triggers and chronic remodeling such as the changes in the sympathovagal balance [33, 34]. Notably, in our paper, we did not find a higher incidence of procedure‐induced AF in the athletic group; a finding that warrants further exploration, particularly in the population of professional endurance athletes [35, 36, 37].

## 5 Limitations

The first limitation of this study is the lack of professional athletes in the examined cohort. Although this limits the generalization of the findings to the professional sports world, the population analyzed is representative of a real‐world population of athletes practicing different disciplines. Furthermore, given that these athletes were not professionals, the duration of DAPT and SAPT was managed in line with current recommendations for the general population (although SAPT was shortened, when possible, in athletes), providing important information about long‐term safety. Furthermore, in this study, we did not establish a tailored DAPT and SAPT approach for the specific population of athletes, and the mean duration of DAPT in the overall population was 1.62 ± 1.5 months. Further long‐term studies are needed to assess the safety and efficacy of alternative default approaches in this specific population.

The study is based on a single‐centre experience; therefore, the present findings cannot be generalized to the entire population of athletes worldwide.

The sample size is relatively small; however, this limitation is offset by a long‐term clinical follow‐up that provides relevant information to the clinical and scientific communities.

We did not provide quantitative echocardiographic data collected during the follow‐up. However, the baseline echocardiographic data demonstrated that none of the participants had a clinically relevant echocardiographic anomaly. Although systolic pulmonary arterial hypertension was not diagnosed or suspected by echocardiography in any of the patients enrolled in this study, right heart catheterization was not performed to estimate pulmonary vascular resistance in patients who underwent ASD closure.

## 6 Conclusions

The percutaneous closure of PFO or ASD is safe and beneficial for athletes who are practicing sports. Awareness of rare complications, such as major or minor bleeding or device embolization, is important. In this study, with a long‐term follow‐up, we demonstrated that the rate of complications and bleeding is rare in athletes undergoing percutaneous closure of PFO and ASD; it does not differ from that of sedentary individuals. Only one case of sports‐related minor bleeding was reported in a cohort of athletes, demonstrating that sports practice does not increase the risk of bleeding, even among athletes engaged in trauma‐risk sports on the SAPT. Further research is needed to confirm these findings in professional athletes and to validate additional strategies for tailoring antiplatelet therapy in this specific population.

### 6.1 Perspective

Although patent foramen ovale (PFO) and atrial septal defect (ASD) percutaneous closure is routinely performed, data on long‐term safety and efficacy in athletes are lacking. In a study with a long‐term follow‐up (> 10 years), we demonstrated that PFO/ASD percutaneous closure is effective and safe in athletes, with no differences between athletes and non‐athletes in the complication rate. Major bleedings do not differ between athletes and non‐athletes, and no sports‐related major bleedings were observed in athletes returning to play, in a long‐term follow‐up of sports practice. Therefore, sports participation after PFO/ASD percutaneous closure does not significantly increase the bleeding risk even in athletes practicing sports at risk of trauma.

As interventional cardiology continues to intersect with elite sports medicine, the long‐term impact of PFO and ASD closure in elite athletes deserves focused investigation. Crucially, the long‐term incidence of atrial fibrillation or other arrhythmias—particularly under chronic high‐output stress—warrants systematic surveillance, given its implications for both athlete safety and performance. This issue is especially pertinent for endurance athletes, such as professional cyclists and marathon runners, whose cardiac physiology is adapted to extreme hemodynamic loads. The biomechanical interaction between intracardiac devices and an athlete's enlarged atrial and ventricular chambers may vary significantly depending on the material composition, shape, and anchoring mechanism of the closure device. Such variability could potentially influence diastolic compliance or atrial function. Therefore, to guide evidence‐based return‐to‐play protocols and clinical management in elite athletes, there is a compelling need for standardized, longitudinal assessment of cardiac mechanics using stress echocardiography or cardiac MRI. Future studies should prioritize athletic subcohorts and integrate both structural and performance endpoints to elucidate the nuanced effects of septal defect closure in this unique population.

## Conflicts of Interest

The authors declare no conflicts of interest.

## Supporting information


**Table S1:** Contact vs. non‐contact sports practiced by the athletic population.

## Data Availability

The data that support the findings of this study are available on request from the corresponding author. The data are not publicly available due to privacy or ethical restrictions.
