# Supplementary material for: Percutaneous Closure of Patent Foramen Ovale and Atrial Septal Defect in Athletes: A Study With a Long‐Term Follow‐Up
Source: Scand J Med Sci Sports. 2025 Aug 8;35(8):e70116. doi: 10.1111/sms.70116 (PMC12333471; doi:10.1111/sms.70116)
Supplement: Supplementary file 1 — Table S1: Contact vs. non‐contact sports practiced by the athletic population. [file SMS-35-e70116-s001.docx]

**Supplementary table 1.** Contact vs non-contact sports practiced by the athletic population

| **Contact sports, no. (%)** | **14 (39)** |
| --- | --- |
| *Football, no. (%)* | 7 (19) |
| *Cycling, no. (%)* | 3 (8) |
| *Rugby, no. (%)* | 2 (6) |
| *Kick boxing, no. (%)* | 1 (3) |
| *Basket, no. (%)* | 1 (3) |
|  |  |
| **Non-contact sports, no. (%)** | **22 (61)** |
| *Running, no. (%)* | 12 (33) |
| *Swimming, no. (%)* | 4 (11) |
| *CrossFit, no. (%)* | 2 (6) |
| *Scuba diving, no. (%)* | 2 (6) |
| *Padel, no. (%)* | 2 (6) |
